# Correction: Hegazy et al. Terazosin Interferes with Quorum Sensing and Type Three Secretion System and Diminishes the Bacterial Espionage to Mitigate the *Salmonella* Typhimurium Pathogenesis. *Antibiotics* 2022, *11*, 465

**DOI:** 10.3390/antibiotics14060529

**Published:** 2025-05-22

**Authors:** Wael A. H. Hegazy, Ibrahim M. Salem, Hadil Faris Alotaibi, El-Sayed Khafagy, Doaa Ibrahim

**Affiliations:** 1 Department of Microbiology and Immunology, Faculty of Pharmacy, Zagazig University, Zagazig 44519, Egypt; 2 Department of Medicinal Chemistry, Faculty of Pharmacy, Suez Canal University, Ismailia 41522, Egypt; dr_ibrahim_m@yahoo.com; 3 Department of Pharmaceutical Sciences, College of Pharmacy, Princess Nourah Bint AbdulRahman University, Riyadh 11671, Saudi Arabia; hfalotaibi@pnu.edu.sa; 4 Department of Pharmaceutics, College of Pharmacy, Prince Sattam Bin Abdulaziz University, Al-kharj 11942, Saudi Arabia; e.khafagy@psau.edu.sa; 5 Department of Pharmaceutics and Industrial Pharmacy, Faculty of Pharmacy, Suez Canal University, Ismailia 41522, Egypt; 6 Department of Nutrition and Clinical Nutrition, Faculty of Veterinary Medicine, Zagazig University, Zagazig 44511, Egypt; doibrahim@vet.zu.edu.eg

## Error in Figure

In the original publication, there was a mistake in Figure 2C as published [1]: a light microscope image of biofilm formation was inserted incorrectly. The corrected Figure 2C appears below. 

In the original publication, there was a mistake in Figure 4A as published [1]: the second panel in Figure 4A was incorrectly labeled as “norepinephrine-treated bacteria”, when it in fact corresponds to “untreated bacteria”. The corrected Figure 4A appears below. 

The authors state that the scientific conclusions are unaffected. This correction was approved by the Academic Editor. The original publication has also been updated.

## Figures and Tables

**Figure 2:**
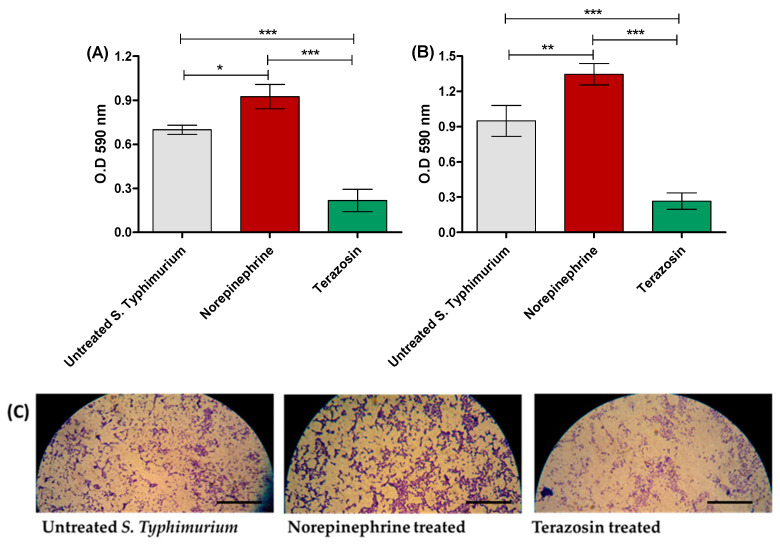
Terazosin diminishes *S.* Typhimurium (**A**) adhesion and (**B**) biofilm formation. Terazosin significantly decreased the bacterial adhesion and biofilm production in comparison to untreated bacteria or norepinephrine-treated bacteria. However, norepinephrine significantly increased bacterial adhesion and biofilm formation. (**C**) Light microscope images of the formed biofilms in the presence of norepinephrine or terazosin. Norepinephrine markedly increased the formed biofilm and terazosin markedly diminished biofilm formation. The experiment was repeated in triplicate, and the data are presented as the mean ± the SD. A one-way ANOVA test, followed by Tukey’s multiple comparison post-hoc test, was employed to attest the statistical significance; *p* < 0.05 was considered significant. *** = *p* < 0.0001; ** = *p* < 0.001; * = *p* < 0.05. Scale bars correspond to 100 μm.

**Figure 4:**
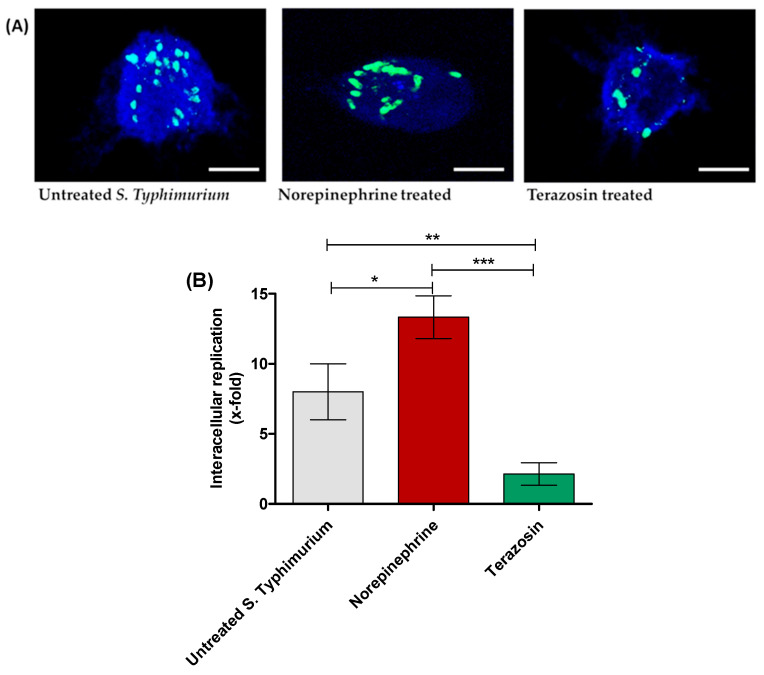
Terazosin diminishes *S.* Typhimurium intracellular replication in raw macrophage cells. (**A**) Microscopic images of *S.* Typhimurium in macrophages. Norepinephrine markedly increased the number of intracellularly replicating bacterial cells and terazosin at the sub-MIC diminished the bacterial intracellular replication in comparison to untreated *S.* Typhimurium. (**B**) The infected macrophages were lysed, and the phagocytosed cell/relative untaken cell percentage and x-fold intracellular replication were calculated. The experiment was repeated in triplicate, and the data are presented as the mean ± the SD. A one-way ANOVA test, followed by Tukey’s multiple comparison post-hoc test, was employed to attest the statistical significance; *p* < 0.05 was considered significant. *** = *p* < 0.0001; ** = *p* < 0.001; * = *p* < 0.05. Scale bars correspond to 100 μm.
